# Neural Networks Trained *via* Reinforcement Learning Stabilize Walking of a Three-Dimensional Biped Model With Exoskeleton Applications

**DOI:** 10.3389/frobt.2021.710999

**Published:** 2021-08-06

**Authors:** Chujun Liu, Musa L. Audu, Ronald J. Triolo, Roger D. Quinn

**Affiliations:** ^1^ Department of Mechanical Engineering, Case Western Reserve University, Cleveland, OH, United States; ^2^ Advanced Platform Technology Center, Louis Stokes VA Medical Center, Cleveland, OH, United States; ^3^ Department of Biomedical Engineering, Case Western Reserve University, Cleveland, OH, United States

**Keywords:** biped stability, gait, neural network, exoskeleton, reinforcement learning

## Abstract

Our group is developing a cyber-physical walking system (CPWS) for people paralyzed by spinal cord injuries (SCI). The current CPWS consists of a functional neuromuscular stimulation (FNS) system and a powered lower-limb exoskeleton for walking with leg movements in the sagittal plane. We are developing neural control systems that learn to assist the user of this CPWS to walk with stability. In a previous publication (Liu et al., Biomimetics, 2019, 4, 28), we showed a neural controller that stabilized a simulated biped in the sagittal plane. We are considering adding degrees of freedom to the CPWS to allow more natural walking movements and improved stability. Thus, in this paper, we present a new neural network enhanced control system that stabilizes a three-dimensional simulated biped model of a human wearing an exoskeleton. Results show that it stabilizes human/exoskeleton models and is robust to impact disturbances. The simulated biped walks at a steady pace in a range of typical human ambulatory speeds from 0.7 to 1.3 m/s, follows waypoints at a precision of 0.3 m, remains stable, and continues walking forward despite impact disturbances and adapts its speed to compensate for persistent external disturbances. Furthermore, the neural network controller stabilizes human models of different statures from 1.4 to 2.2 m tall without any changes to the control parameters. Please see videos at the following link: 3D biped walking control.

## 1 Introduction

Our group is developing a cyber-physical walking system (CPWS) with a hybrid neuromuscular/motor power source to restore stable walking in individuals with paralysis caused by spinal cord injury (SCI) (Nandor et al., 2021). The CPWS includes a human with SCI using a neuromuscular stimulation system, a powered exoskeleton, and a sensor-based control system that activates the otherwise paralyzed muscles and actuates joint motors. The human muscles are the primary motivator, and the exoskeleton’s motors are activated on an as-needed basis. The goal for the CPWS is to generate a natural gait and stable walking. Whereas most existing exoskeletons are stabilized by the human user exerting upper body effort with a walker, canes, or crutches, our goal is for the system to be primarily self-stabilizing and allow the user to walk upright functionally and for more satisfying social interactions. The control problem is challenging because it involves not only bipedal walking control, but also requires careful human-machine interaction design for safety and efficient movement. This paper describes a part of the CPWS control system that will assist in stabilizing the system.

The need for stabilizing exoskeletons has been addressed by other researchers. There are many bipedal robots that demonstrate stable walking and even acrobatic abilities (Guizzo, 2019). In some ways, controlling an exoskeleton is similar to controlling a bipedal robot. Thus, the concepts and methods related to bipedal walking control can be utilized in exoskeleton control, particularly since exoskeletons have previously been modeled as serially linked mechanisms (Kazerooni et al., 2005). Kazerooni et al. (2005) developed a controller for an exoskeleton that augmented able-bodied human performance. It increased the closed-loop system sensitivity to its wearer’s forces and torques without any conscious intervention from the wearer. However, the controller was not robust to parameter variations and therefore required a good dynamic model of the system. In this work, we achieve robustness to parameter variations by using a learning-based algorithm. A similar concept was used in Kong and Jeon (2006). They proposed a new adaptive sliding mode repetitive learning control strategy for upper-limb exoskeletons that were robust to unknown dynamics and external disturbances. Kong and Jeon (2006) proposed a tendon-driven exoskeletal assistive device. However, it was for movements in the sagittal plane and required the use of a walker. Li et al. (2020) proposed an algorithm based on the zero moment point (ZMP) to modify the gait generated through human walking synergy for paraplegic patients but required the use of bilateral canes, or crutches. Campbell et al. (2020) combined virtual constraint control with a velocity-modulated dead-zone to ensure the stability of a walking model. Zhang et al. (2018) presented a balance controller based on the extrapolated center of mass concept for maintaining walking stability.

We chose to use artificial neural networks (ANNs) with reinforcement learning (RL) techniques to stabilize the CPWS because of the powerful new tools that are available and because of previous successes in applying them to stabilize bipedal walking. Traditional trajectory optimization-based control suffers from computational cost and cannot resist large disturbances. Also, the resulting gait depends on the chosen objective function (Ackermann and Van den Bogert, 2010). For bipedal gait control problems, the RL method must be able to handle continuous input and output. Several RL algorithms satisfy this requirement, such as Deep Deterministic Policy Gradient (DDPG) (Silver et al., 2014), Proximal Policy Optimization (PPO) (Schulman et al., 2017), Covariance Matrix Adaptation (CMA) (Hansen and Ostermeier, 1996), and Neuro-Evolution of Augmenting Topologies (NEAT) (Stanley and Miikkulainen, 2002). NEAT and CMA are especially good for optimizing problems with a small set of parameters. Song and Geyer (2015) uses CMA to optimize a biped controller with 82 parameters. However, many more parameters are usually needed for a densely connected neural network. DDPG and PPO can be used to train an agent parameterized by a neural network. We chose to use PPO because of its stability characteristics.

Artificial neural networks have many advantages, especially when the underlying system dynamics are unclear or difficult to model. One disadvantage, however, is that the network training process is data thirsty. This can be mitigated by using simulation to pre-train the network. Typically, the simulation needs to run 10^7^ to 10^9^ steps for the network to converge to a good result (Nagabandi et al., 2018). Thus, it is difficult to directly implement reinforcement learning techniques on a mechanical system. Reference motions can be used to guide and facilitate the training process (Peng et al., 2017; Haarnoja et al., 2018; Abdolhosseini et al., 2019; Yin et al., 2007), but it still needs hundreds of thousands of training steps.

In this paper, we report on a new controller that solves the above problems and expands on our previous work. Our previous paper Liu et al. (2019) used a deep deterministic policy gradient (DDPG) neural network to predict the ideal foot placement for a two dimensional biped model to maintain stable walking despite external disturbances. We found that this approach was not readily translated to three dimensions. Here, we report on our new learning-based controller for stance and swing of three dimensional bipeds; it is less model sensitive, is robust to impulsive and persistent disturbances, and is relatively fast to train.

## 2 Method

In this paper, we first developed a core control system based on a simplified dynamic model. Then we used its outputs to train an artificial neural network (ANN) controller using a reinforcement learning algorithm. The core control system was developed using classical control methods, and is strongly dependent on the exact mechanical parameters of the model. The RL neural network controller was found to be superior because it is robust to model parameters. With the ANN controller, a different person could use the exoskeleton without changing the controller parameters.

The core control system was based on classical control methods and a reduction in the complexity of the system was essential for its success. To reduce the dimensions of the problem, Alibeji et al. (2015) used principal component analysis, whereas Chevallereau et al. (2009) used virtual constraints for a similar reason. We reduced the dimensions and complexity of the control system by developing separate controllers for the swing and stance phases. To simplify the problem, we assumed that the swing leg’s reaction forces have little effect on the torso at the walking speed of the CPWS (1 m/s), and our subsequent results justify this assumption. Thus, we divided the control task into two parts: The torso stability task for the stance leg and the optimal foot placement task for the swing leg. The core control system stabilized the biped and rejected impacts and persistent disturbances, but it was not robust to changes in the model.

A neural network was developed and trained using reinforcement learning, starting with data from the core controller. Two ANN controllers were developed: A “local” controller and a “global” controller. The local controller was divided into two sub-controllers, one for swing and one for stance. The advantage of this method is that the action space is small for each sub-controller, so the neural net converged faster. But the disadvantage is that we needed to alternately freeze the parameters of one neural net and train the other one so that they can be better coordinated with each other. Although the stance leg controller is minimally dependent on or entirely independent of the swing foot placement controller based on this assumption, the reverse is not true. The foot placement controller takes both the motion of the swing leg and the stance leg into consideration, and the reinforcement learning optimization process also takes this into consideration. Thus, the parameters for the local neural net swing leg foot placement controller were optimized after the local stance controller was tuned such that the neural net learned the effect of the stance controller on the foot placement controller.

The global controller was trained for the system as a whole rather than separating it into swing and stance controllers. We compared the convergence rates and the performances of the local and global controllers and found that the local controller converged faster but did not perform as well as the globally trained controller.

## 3 Biped Model

**FIGURE 1:**
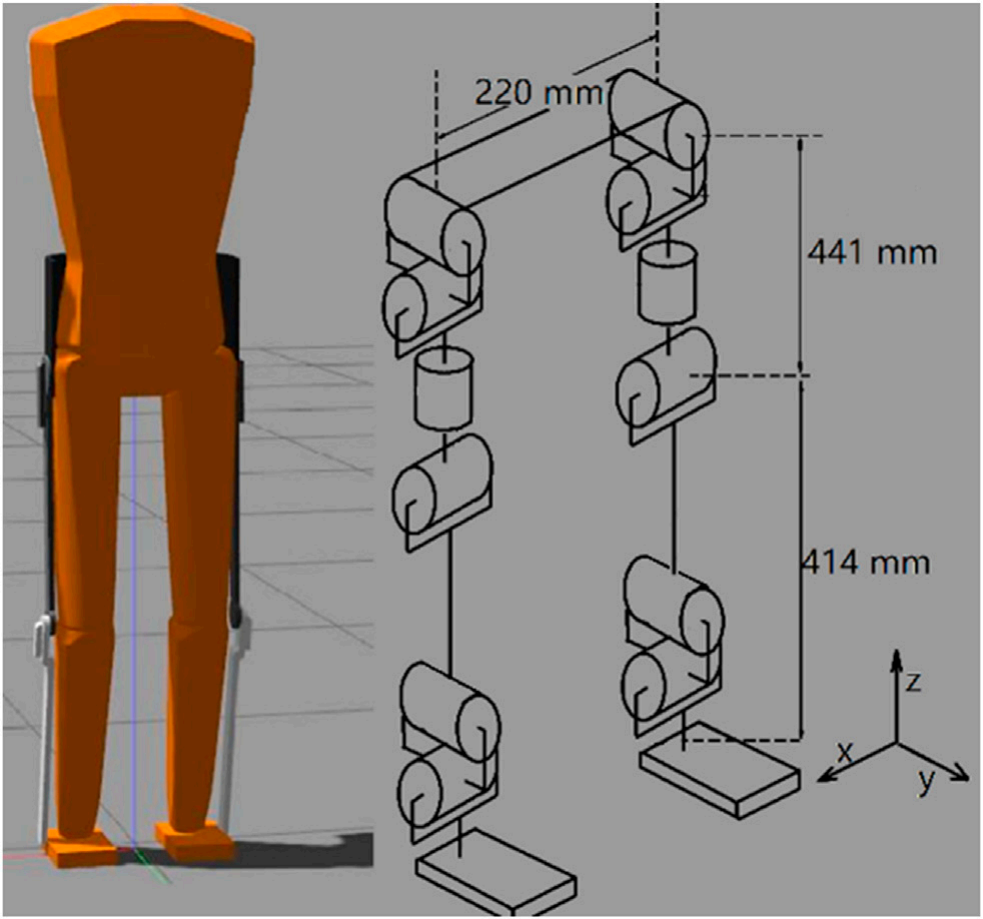
GAZEBO simulation model. Each leg has six degrees of freedom: Three at the hip, one at the knee, and two at the ankle.

The bipedal model was developed in the Gazebo simulation environment (Koenig and Howard, 2004), which can provide a high-performance 3-D physics simulation. The default physics engine used by GAZEBO is ODE (Open Dynamics Engine), which was used in this work. It also supports other physics engines, such as Bullet, Dynamic Animation and Robotics Toolkit (DART), and Simbody. GAZEBO is popular in robotics and was used in the DARPA Robotics Challenge. It is often used with ROS (Robot Operating System), a popular API that provides commonly used tools for robotic applications. The simulated robot can be easily controlled through the GAZEBO-ROS interface, as was done here. The model has a total of 12 joint DOF (degrees of freedom), 6 DOF on each leg, as shown in Figure 1: Three hip joint DOF, one knee joint, and two ankle joint DOF. Individual segment masses and lengths are proportioned based on human studies for a 1.8 m tall male (Yu-Cheng et al., 2004). Mass and inertia are added to realistically represent an exoskeleton and its associated electronics as shown in Table 1. Joint limits are set to allow the full range of motion (Zoss et al., 2005). The mass moment of inertia and the collision models are simplified as rectangular parallelepipeds. Mass and inertia properties for a particular user can be measured through experiments (Ghan et al., 2006). The torso degrees of freedom are assumed fixed by the exoskeleton corset brace, as is done on the CPWS.

**TABLE 1:** model specification.

Link name	Mass (kg)	Dimension (mˆ3)	Offset (m)
Torso	50.85	0.36*0.18*0.72	(0,0,0)
Thigh	7.5	0.1*0.1*0.441	(0,0,0)
Shank	4.4875	0.1*0.1*0.414	(0,0,0)
Foot	1.5	0.11*0.17*0.03	(0,0,0)
Backpack	5	0.36*0.1*0.18	(0,−0.1,0)
Exoskeleton thigh	2	0.1*0.1*0.441	(0,0.05,0)
Exoskeleton shank	2	0.1*0.1*0.414	(0,0.05,0)

## 4 Development of a Core Controller *via* Classical Control Methods

The “core controller” was developed using classical control methods and then used to create data to initialize the neural network controllers to start the reinforcement learning process.

### 4.1 Stance Leg Controller

The stance leg controller is intended to stabilize the torso during the single-limb support phase. It is designed such that the torso’s pitch angle and rotational velocity are small, and its vertical acceleration and velocity are negligible. This greatly simplifies swing foot placement control by representing the single support stance limb as a double inverted pendulum. For the stance leg controller development, we assume:

1) The knee joint angle and its angular velocity are small, so the entire leg can be treated as a single link.
2) The foot/ground friction is large enough so that there is no slipping.
3) The reaction force resulting from the motion of the swing leg is small.
4) The control torque in the ankle is small enough to not cause the foot to rotate about the toe.

The equation of motion of the system can be derived from Lagrange’s equation:

$$\frac{d}{dt}\left(\frac{\partial \left(T-V\right)}{\partial \dot{q}}\right)-\frac{\partial \left(T-V\right)}{\partial q}=Q$$ (1)

In matrix form:

$$Dq..+C\dot{q}+G=Q$$ (2)

*q* = [*q*_1_, *q*_2_, *q*_3_, *q*_4_] is the set of joint angles representing hip-x (Abduction/Adduction), hip-y (Flexion/Extension), ankle-x (Dorsiflexion/Plantarflexion) and ankle-y (Eversion/Inversion). Hip-z (Lateral/Medial) is locked during stance control. *Q* = [*Q*_1_, *Q*_2_, *Q*_3_, *Q*_4_] is the control torque that acts on joints 1, 2, 3, and 4. *T* and *V* are the kinetic and potential energies of the system. The goal is to find the control torque *Q* to drive the torso angle *P* and its velocity $\dot{P}$ to zero. The torso angle *P* is defined as the angle between the torso *z*-axis and the global *z*-axis. Assume a unit vector in the *z*-direction, *k* = [0,0,1]^*T*^, is attached to the torso. Then the vector expression transformed into the global frame is:

$$k^{\prime }\left(q\right)=R_{{\text{ankle }}_{x}}R_{\text{ankley }}R_{hip_{x}}R_{hip_{y}}k$$ (3)

Where $R_{{ankle}_{x}},R_{{ankle}_{y}},R_{{hip}_{x}},R_{{hip}_{y}}$ are rotational transformation matrices associated with each joint. The subscript x and subscript y designate rotation about the x or *y* axis, respectively. Then, the angle between the *k*′ and world *z*-axis can be calculated as:

$$P\left(q\right)=\mathrm{acos}\left(\left[0,0,1\right]\cdot k^{\prime }\left(q\right)\right)$$ (4)

By taking derivatives of *P*, we can calculate the velocity and acceleration of the angle:

$$\dot{P}=\frac{\partial P\left(q\right)}{\partial q}\dot{q}$$ (5)

$$\overset{..}{P}=\frac{\partial^{2}P\left(q\right)}{\partial q^{2}}\dot{q}+\frac{\partial P\left(q\right)}{\partial q}\overset{..}{q}$$ (6)

A constraint equation is imposed on the system so that it will drive the torso angle *P* to zero:

$$\overset{..}{P}+k_{p}P+K_{v}\dot{P}=0$$ (7)

*Q*_1_ and *Q*_2_ are control torques on the ankle. These are set to 0 because the ankle is assumed to be an un-actuated joint. One constraint equation is not enough, and there are infinite solutions. Thus, we instead consider the x and y components of vector *k*′. To minimize the angular rotations of the torso, we desire the x and y components of *k*′ to go to 0 at the same time. But in this way, we must assume that the torso angle P is smaller than *π*/2. Otherwise, when we decrease the x and y components of the vector, the angle *P* will increase instead of decrease. The case where angle *P* is larger than *π*/2 does not happen during normal walking. The velocity and acceleration of the x and y components of *k*′ can be calculated in the same way:

$$\begin{array}{c} X=\left[1,0,0\right]\cdot k^{\prime }\left(q\right)\\ \dot{X}\left(q,\dot{q}\right)=\frac{\partial X\left(q\right)}{\partial q}\dot{q}\\ \overset{..}{X}\left(q,\dot{q},q..\right)=\frac{\partial^{2}X\left(q\right)}{\partial q^{2}}\dot{q}+\frac{\partial X\left(q\right)}{\partial q}q..\\ Y=\left[0,1,0\right]\cdot k^{\prime }\left(q\right)\\ \dot{Y}\left(q,\dot{q}\right)=\frac{\partial Y\left(q\right)}{\partial q}\dot{q}\\ Ÿ\left(q,\dot{q},q..\right)=\frac{\partial^{2}Y\left(q\right)}{\partial q^{2}}\dot{q}+\frac{\partial Y\left(q\right)}{\partial q}q.. \end{array}$$ (8)

We design the acceleration so that *X* and *Y* are stable and converge to 0 so that the torso remains vertical. Thus, negative position and velocity feedback is used:

$$\begin{array}{c} {X..}_{d}=-k_{px}X-K_{vx}\dot{X}\\ {Y..}_{d}=-k_{py}Y-K_{vy}\dot{Y} \end{array}$$ (9)

where *K*_*p*_ and *K*_*v*_ are feedback gains for position and velocity. By choosing different gain values, we can manipulate the behavior of these second-order systems. Desired response time and overshoot values can be achieved by using the pole placement method. A large damping value is preferred because oscillation of the torso will decrease the stability of the foot placement controller.

In summary, the following set of equations can be solved for the joint accelerations.

$$\left[\begin{array}{c} D_{u}\\ \frac{\partial X}{\partial q}\\ \frac{\partial Y}{\partial q} \end{array}\right]q..+\left[\begin{array}{c} C_{u}\\ \frac{\partial^{2}X}{\partial q^{2}}+k_{v}\frac{\partial X}{\partial q}\\ \frac{\partial^{2}Y}{\partial q^{2}}+k_{v}\frac{\partial Y}{\partial q} \end{array}\right]\dot{q}+\left[\begin{array}{c} G_{u}\\ k_{p}X\\ k_{p}Y \end{array}\right]=0$$ (10)

*D*_*u*_, *C*_*u*_, *G*_*u*_ are the inertia, centrifugal-Coriolis, and gravity terms for the un-actuated joints. Then the control torques can be solved using Eq. 2. Next, a constraint on the ground reaction force is imposed so that there is no foot/ground slip, and the foot stays on the ground for the entire stance phase. The constraints are expressed as:

$$\left\{\begin{array}{c} F_{x}^{2}+F_{y}^{2}<\mu^{2}F_{z}^{2}\;\\ F_{z}>0\; \end{array}\right.$$ (11)

A solution that satisfies these constraint was found using optimization. The variables that need to be optimized are control gains *k*_*p*_ and *k*_*v*_ in Eq. 9 for *X* and *Y*. The time domain solution can be expressed in terms of a matrix exponent.

$$\begin{array}{cc} X¯ & =\left[\begin{array}{c} X\\ \dot{X} \end{array}\right]=e^{A_{X}t}{X¯}_{0}\\ A_{X} & =\left[\begin{array}{cc} 0 & 1\\ -k_{px} & -k_{vx} \end{array}\right] \end{array}$$ (12)

Y¯ equations are similar to Eq. 12 for X¯. The objective function *J* is the integral of the square sum of the future error:

$$J=\int_{0}^{T}{\left(e^{A_{X}t}{X¯}_{0}\right)}^{T}e^{A_{X}t}{X¯}_{0}+{\left(e^{A_{Y}t}{Y¯}_{0}\right)}^{T}e^{A_{Y}t}{Y¯}_{0}dt$$ (13)

**FIGURE 2:**
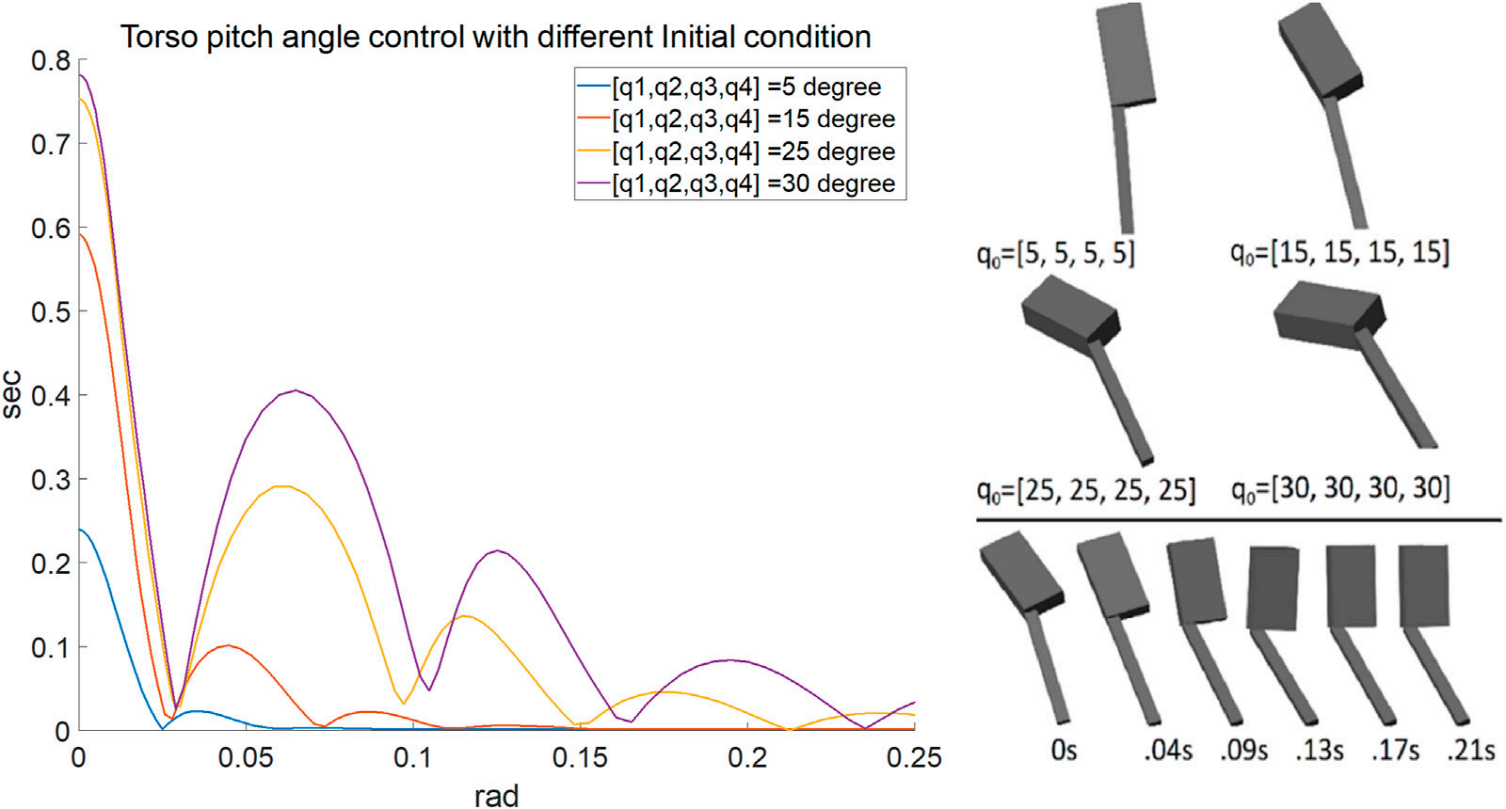
Left shows the time response of the torso angle for different initial conditions. Right graphically shows the different initial conditions and a snapshot of the process of the torso angle stabilization with 15 degrees initial conditions for each joint. The upper rectangular block is the torso, and the lower bar is the leg. Note that the knee is locked.

This optimization problem needs to be solved at every time step. It takes approximately 0.02–0.05 s using the MATLAB optimization toolbox function “fmincon ()”. This was found to consume much computational power and reduce the control frequency. So instead of performing this optimization, the difference between the generated joint acceleration (from Eq. 10) and zero torque joint acceleration were used to limit the torque output of the controller. In a loop, if the constraint is not met, then the control output is reduced:

$$\overset{..}{q}\leftarrow q..+\lambda \left(\overset{..}{q}-{q..}_{0}\right),\;0<\lambda <1$$ (14)

q.. is the zero-torque joint acceleration, and *λ* is the decay factor. Figure 2 shows the torso angle control in single limb support phase.

### 4.2 Foot Placement Controller

After the torso angle is stabilized by the stance leg controller, if the torso angular velocity is small, then the motion of the biped during the swing can be approximated by a single 3-D inverted pendulum. In Kajita et al. (2001), a 3D Linear Inverted Pendulum Model was developed. If the mass center motion plane is parallel with the ground, then the pendulum’s time-invariant orbital energy can be calculated by integrating the equations of motion along the *x* and *y* axis with respect to time.

$$\begin{array}{cc} E_{y} & =\frac{1}{2}{\dot{y}}^{2}-\frac{g}{2z}y^{2}\\ E_{x} & =\frac{1}{2}{\dot{x}}^{2}-\frac{g}{2z}x^{2} \end{array}$$ (15)

The phase plane of the trajectory shows that if the orbital energy is greater than 0, then the trajectory will cross the zero position, and if the orbital energy is less than 0, the trajectory will not. If the orbital energy is equal to 0, then the trajectory will come to rest at a saddle point, also known as capture point (Pratt et al., 2006). So, for walking of a 3-D biped in the *y* direction, for example, then *E*_*y*_ must be greater than 0, and for stability in lateral motion, it is preferred that *E*_*x*_ is less than 0 so that the COM can oscillate between the two feet. From Eq. 15, the initial position can be calculated for the foot placement.

$$\begin{array}{c} y=\sqrt{\left(\frac{{\dot{y}}^{2}}{2}-E_{y}\right)\frac{2z}{g}}\\ x=\sqrt{\left(\frac{{\dot{x}}^{2}}{2}-E_{x}\right)\frac{2z}{g}} \end{array}$$ (16)

The calculated initial conditions for foot placement from this model can only generate several steps of stable walking before falling down because of the disturbances from the torso. The model has several restrictions: First, the mass center must move in a plane that is parallel to the ground. Second, the mass center is concentrated around the tip of the pendulum. Third, there is no control input. In practice, it is difficult to satisfy all these constraints. Brasseur et al. (2015) bound the nonlinear term resulting from the COM vertical motion to a region where the linear controller still generates a dynamically feasible solution. We added modified terms and coefficients to compensate the difference in modeling:

$$\begin{array}{c} x=\pm d+\mathrm{clip}\left(\dot{x},1,-1\right)\cdot \sqrt{\left(\frac{{\dot{x}}^{2}}{2}-E_{x}\right)\frac{2z}{g}}\;*\left(1+0.1|P|\right)\\ y=\mathrm{clip}\left(\dot{y},1,-1\right)\cdot \sqrt{\left(\frac{{\dot{y}}^{2}}{2}-E_{y}\right)\frac{2z}{g}}\;*\left(1+0.1|P|\right) \end{array}$$ (17)

Where d is the horizontal distance between the hip joint and the center of mass. The clip coefficient regulates the sensitivity of the velocity influence. The coefficient 1 + 0.1|*P*| compensates for the influence from the torso angle. Initial conditions for the center of mass are transformed into foot placement by calculating the forward kinematics. The swing leg trajectory can be generated by inverse kinematics relative to the mass center. In our approach, the inverse kinematics for x and y are relative to the mass center, and z is relative to the global reference frame. In this way, the z component of the foot displacement will not be affected by sudden movements of the mass center and, thus, the ground contact is more controllable. The position of the foot *X*_*f*_ can be expressed as:

$$\left[\begin{array}{c} 0\\ 0\\ z_{c} \end{array}\right]+R\cdot \left(X_{c-h}+F\left(q\right)\right)=X_{f}$$ (18)

Where *z*_*c*_ is the z component of the mass center in global coordinates and *R* is the rotation matrix from global coordinates to body-fixed coordinates. *F*(*q*) is a vector of the Cartesian coordinates of the foot relative to the hip expressed as a function of the joint angles *q*. *X*_*f*_ is the foot coordinates partially relative to COM in global coordinates. *X*_*c*−*h*_ is a vector from the mass center to the hip joint in body-fixed coordinates:

$$X_{c-h}=X_{GC-hip}-X_{GC-COM}$$ (19)

*X*_*GC*−*hip*_ is a vector from the torso’s geometric center to the hip joint in the body-fixed coordinates, and *X*_*GC*−*COM*_ is a vector from torso’s geometric center to the mass center in body-fixed frame. *X*_*GC*−*COM*_ is a function of the joint angles *q*. Taking the time derivative of both sides of Eq. 18:

$$\left[\begin{array}{c} 0\\ 0\\ {\dot{z}}_{c} \end{array}\right]+\dot{R}\cdot \left(X_{c-h}+F\right)+R\cdot \left({\dot{X}}_{c-h}+\dot{F}\right)={\dot{X}}_{f}$$ (20)

$\dot{R}$ is a function of Euler angles (*α*, *β*, *γ*) and their time derivatives.

$${\dot{X}}_{c-h}={\dot{X}}_{GC-hip}-{\dot{X}}_{GC-COM}=-{\dot{X}}_{GC-COM}=-J_{GC-COM}\dot{q}$$ (21)

*J*_*GC*−*COM*_ is the system Jacobean:

$$J_{GC-COM}=\frac{\partial X_{GC-COM}}{\partial q}$$ (22)

*J*_*GC*−*COM*_ can be expressed as [*J*_*GC*−*COM*−*l*_, *J*_*GC*−*COM*−*r*_], and $\dot{q}$ can be written as $\left[\begin{array}{c} {\dot{q}}_{l}\\ \dot{q_{r}} \end{array}\right]$. The subscript *”l”* and*”r”* represent the left and right leg, respectively. Then:

$${\dot{X}}_{c-h}=-J_{GC-COM}\dot{q}=-J_{GC-COM-l}{\dot{q}}_{l}-J_{GC-COM-r}{\dot{q}}_{r}$$ (23)

And

$$\dot{F}=J_{k}{\dot{q}}_{l}\text{ or }\dot{F}=J_{k}{\dot{q}}_{r}$$ (24)

Depending on which leg is the swing leg. *J*_*k*_ is the foot position Jacobian for leg kinematics. For example, if the left leg is the swing leg, then substituting equations:

$$\left[\begin{array}{c} 0\\ 0\\ {\dot{z}}_{c} \end{array}\right]+\;\;\dot{R}\;\;\cdot \left(X_{GC-hip-l}-X_{GC-COM}+F_{l}\right)+R\;\;\cdot \left(-J_{GC-\mathrm{COM}-r}{\dot{q}}_{r}+\left(J_{k}-J_{GC-\mathrm{COM}-l}\right){\dot{q}}_{l}\right)=\dot{X_{f}}$$ (25)

And using inverse kinematics, the left leg joint velocity can be calculated:

$${\dot{q}}_{l}={\left(RJ_{k}-RJ_{GC-\mathrm{COM}-l}\right)}^{-1}\left({\dot{X}}_{f}-\left[\begin{array}{c} 0\\ 0\\ {\dot{z}}_{c} \end{array}\right]-\dot{R}\cdot \left(X_{GC-hip-l}-X_{GC-COM}+F_{l}\right)+RJ_{GC-\mathrm{COM}-r}{\dot{q}}_{r}\right)$$ (26)

This equation is then integrated to find the desired joint angles.

## 5 Developing an Artificial Neural Network Controller and Training it With Reinforcement Learning

As shown in the results below, the core controller derived in Section 4 stabilized the biped for which it was designed. However, it was found to be brittle. It became unstable with even a small change in biped parameters. Thus, to create a more robust controller, an ANN was designed and trained using reinforcement learning starting with data from the core controller.

**FIGURE 3:**
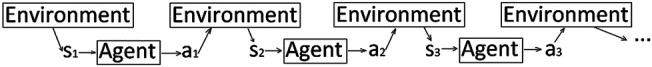
Interaction between a reinforcement learning agent and the environment.

The neural networks for enhancing the core controller need to be able to work with continuous inputs and outputs. There are many reinforcement learning (RL) techniques that can be used to optimize a neural net in such a case. Many are gradient-based and require large samples of trajectories. The training algorithm used in this work is called Proximal Policy Optimization (PPO) (Schulman et al., 2017). It is a popular off-policy gradient-based optimization method. PPO is the default learning algorithm for Open-AI because it is efficient compared to many on-policy stochastic policy gradient methods, and it is straightforward to implement compared to its full version: Trust Region Policy Optimization (TRPO) (Schulman et al., 2015). For a policy gradient method, an agent interacts with its environment, observes its state *s*, and then outputs action *a* according to policy *π*_*θ*_, and then the agent will move to the next state according to action *a* and so on as shown in Figure 3. A trajectory *τ*(*s*_1_, *a*_1_, *s*_2_, *a*_2_, *s*_3_, *a*_3_…) of state and action can be recorded. The probability for *τ* is

$$p\left(\tau \right)=p\left(s_{1}\right)p_{\theta }\left(a_{1}|s_{1}\right)p\left(s_{2}|a_{1},s_{1}\right)p_{\theta }\left(a_{2}|s_{2}\right)p\left(s_{3}|a_{2},s_{2}\right)\ldots$$ (27)

*p*_*θ*_ represents the possibility for the agent to output a certain action given the state. This possibility is controlled by the policy parameter *θ*. The goal is to train this agent so that it will have a high possibility to output the action that can lead to a larger reward. The policy gradient method is an on-policy method, meaning that the data used to calculate the gradient must be gathered by the current policy. Otherwise, a different policy will give a different distribution, and the sampled gradient will not approximate the true gradient. Thus, every time the policy parameter is updated, all the data collected previously will be outdated and cannot be used in the future. This results in the policy gradient method spending most of its computational time collecting data rather than training.

PPO uses importance sampling to mitigate this problem (Schulman et al., 2017). Importance sampling allows one to calculate the expectation of a distribution *p* from a different distribution *q*. However, this expression becomes more difficult to approximate when the difference between the two distributions is large. So, in the PPO algorithm, a KL divergence is added to the objective function to measure the difference between distributions (Goldberger et al., 2003). This KL divergence is used to penalize large differences between distributions. By using this approach, the data can be used to update the parameters several times. PPO can also use a modified surrogate objective *L* ^*CLIP*^(*θ*) to limit the step size during a trust-region optimization update.

As mentioned previously, the goal is to develop a neural network trained using reinforcement learning initialized with data from the core control system. But the structure of the core control system separates the full biped action control into two parts: swing leg control and stance leg control. On the one hand, this reduces the difficulty of the design of each controller. On the other hand, it increases the training difficulty for the reinforcement learning algorithm. Two different methods of implementing the learning algorithm are used in this work and the results are compared.

### 5.1 Local Reinforcement Learning

In the first method, called the “local” method, there are separate neural networks for each action control task: A foot placement controller neural network and a stance leg controller neural network. Because the control task was divided into two parts, the optimization task was eased as compared to trying to optimize a single more complex control system for the entire interconnected dynamic system. Thus, we could find sufficient RL policies for each of these two control problems with fewer iterations.

For the purpose of control, the gait cycle was divided into swing, double-support, stance, and toe-off (we treat the toe-off as a gait phase). Except for the double-support phase, the timing of the phase switching was controlled by feedback from contact sensors at the foot and the output of the controllers. The duration of the double support phase was linearly related to the torso velocity. Simulated experiments showed that this intuitively derived linear relationship was sufficient to represent the system. However, in future work, it could also be optimized using a neural net.

Toe-off can be achieved in two ways. One is to generate a trajectory for the foot using inverse kinematics for the target joint angle controller to lift the foot. The other method is to disable the knee target joint angle controller and apply a direct reflexive torque to flex the knee. This is necessary because the disturbance caused by toe-off often causes the swing foot to hit the ground when the foot does not follow the designed trajectory, and a target angle controller will make the knee joint stiff. It is inefficient and may destabilize the system. Flexing the knee allows the swing foot to move forward freely without the knee pushing the foot against the ground. In normal walking, when there are no disturbances, the trajectory following method is used. But, when a premature foot contact is detected, the controller switches to the reflexive method.

The simulations proceeded as follows. The foot placement controller neural network was active once every footstep. The input vector included torso linear and angular velocities in the *x* and *y* directions, torso angle, torso height, and which leg (swing leg) it should control at that instant. The outputs were the target hip joint angle in the *x*-axis, which controlled how far the biped should step, and the duration of the step, which determined how fast the joint should rotate, and the timing of the knee extension. The stance leg controller neural network was active at 50 hz during stance. The input vector to the controller was the same as the foot placement controller plus additional stance leg joint angle and joint velocity information. The output was the hip joint velocity of the stance leg. The reason to use target joint velocity control is that it can improve policy performance and learning speed (Peng and van de Panne, 2017). The environmental reward function used for the foot placement controller neural network training was simply the forward travel distance of the biped, and the reward for the stance leg control controller neural network was the norm of the torso angle and angular velocity vector. Each episode ended when the mass center height was below the threshold or the travel distance reached a preset maximum value.

After the foot placement controller neural network was optimized, its parameters were frozen, and we then optimized the stance leg controller neural network. The optimization of the two neural networks changed the dynamics of both the stance leg controller and the foot placement controller. Thus, it was found useful to iterate this process so the neural network in each controller could adapt to changes and synergize better with the other. We have found that this training loop was prone to converge because, while the foot placement controller is strongly influenced by the stance controller, the stance leg controller depends little on the foot placement controller. Also, if the simulation displayed some unrealistic behavior due to the numerical solver in Gazebo, then the entire trajectory collected in that episode was not used in training.

### 5.2 Global Reinforcement Learning

In the second method, called the “global” method, only one neural network is trained, and it controlled both the swing leg and the stance leg. The action space was much larger compared to the local method. This neural network was run at 50 hz. The state input was a series of state vectors consisting of torso position and velocity, mass center relative position and velocity, joint angle and velocity, left and right contact sensors, and the output from the core controller. The state also included a target coordinate and the target speed. The input state series allowed the neural net to perceive some events that are hard for a single timestamp state input to represent, such as the ground contact and foot slipping. The length of the input series was set to 5 so that a total of 0.1 s of motion was recorded in the state series in a 50 hz control loop. This is similar to a human’s 0.15 s reaction time for a touch stimulus. The environmental reward was the error norm of current torso position to the target coordinate and current speed to target speed. The output of the neural network was stance leg joint velocity (hip-x, hip-y, knee) and x, y and z coordinates of the predicted foot placement for the swing leg. The final output of the control system was the summation of the output from the neural net and the core controller. At the beginning of the training, the neural net was initialized to output zero means and small variances so that the total output was similar to the output of the core controller. Thus, the agent started its exploration near an optimal point. After the first parameter update of the neural net, the agent departed from the initialized parameters, and the stable gait provided by the core controller was no longer imposed. After some iterations, the agent optimized the policy and generated a stable gait. The RL-trained neural network controllers were much more robust to changes in the model’s mechanical parameters than the core controller, as can be seen in the examples in the results section.

**FIGURE 4:**
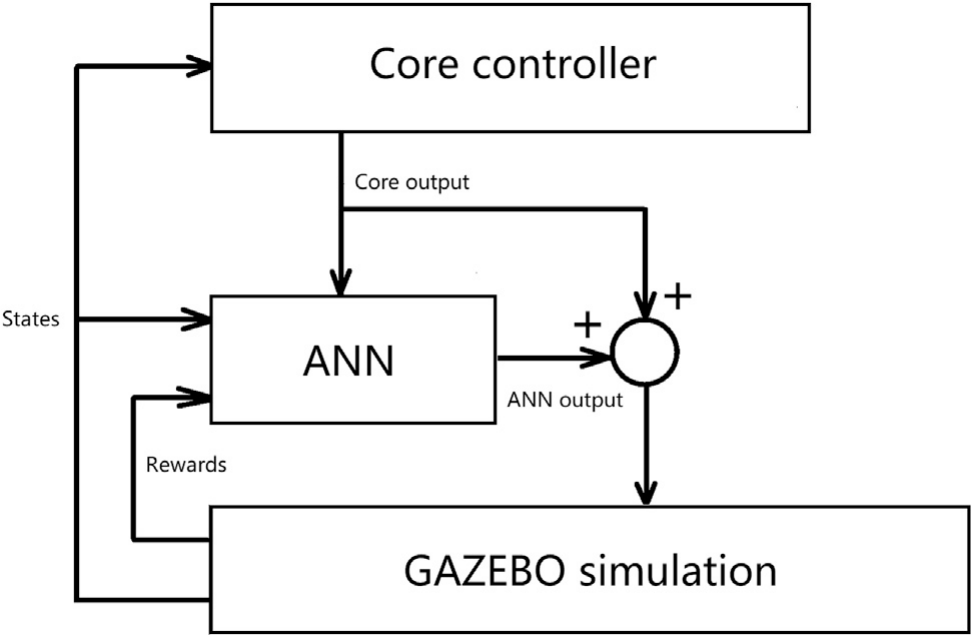
The relationship between the core controller and the ANN. The output from the core controller is both input to the ANN controller and combined with the ANN output and then input as control commands to the biped simulation in GAZEBO.

**FIGURE 5:**
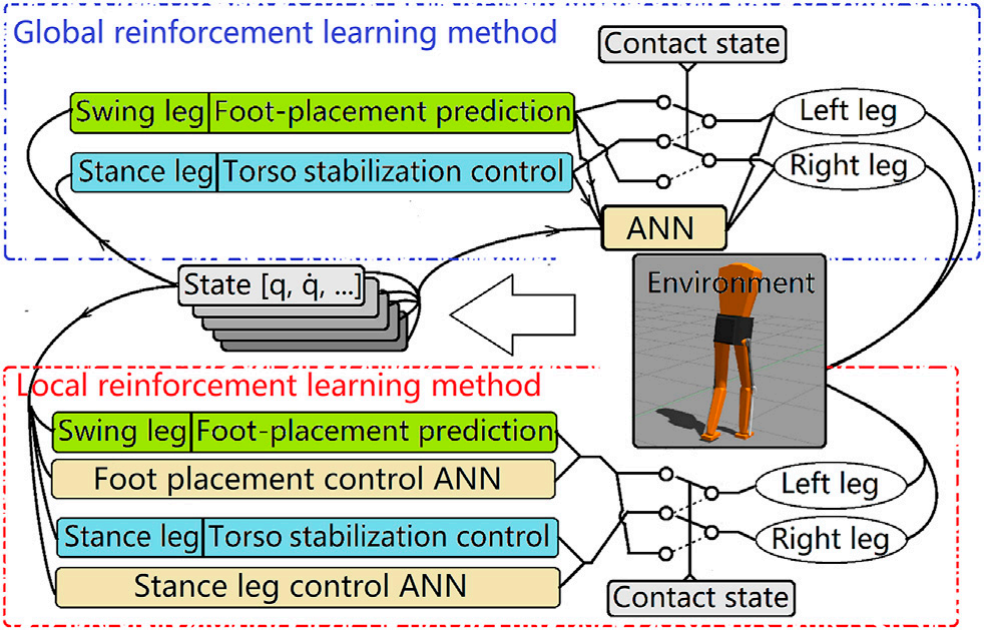
Two different reinforcement learning frameworks. In global reinforcement learning method **(upper)**, outputs from two core controllers (stance leg controller and foot placement controller) are checked and modified by the same neural network. The inputs to the neural net are a series of biped states and the raw output from the core controller. The local reinforcement learning method has separate neural networks for the stance leg and the foot placement controllers. The switching of the stance leg and the swing leg is controlled by the contact sensor.

**FIGURE 6:**
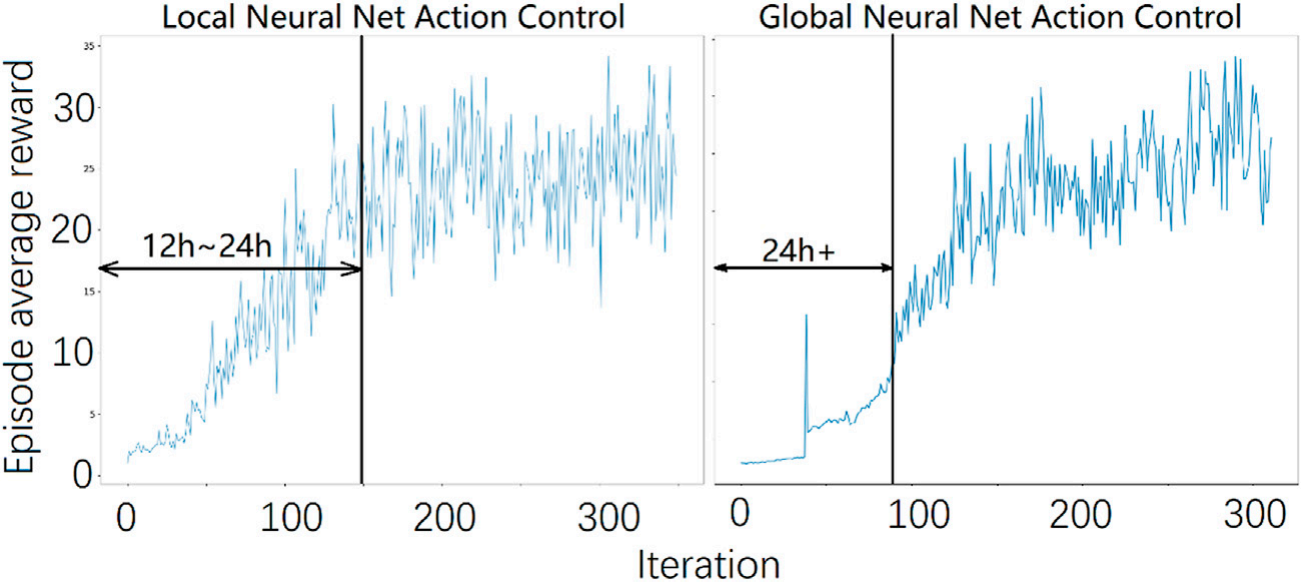
The average episode reward during training. A stable gait emerged at approximately 150 iterations or 15,000 walking steps for the local reinforcement learning method and about 100 iterations or approximately 500,000 steps for the global local reinforcement learning method.

Figure 4 shows the relationship between the core controller and the ANN. Figure 5 summarizes the control flow of the two different methods. The core controller for both methods and the policy in the local reinforcement learning method use only the current state of the biped. The global reinforcement learning method policy uses the current and a series of past states of the biped as input. In the global reinforcement learning method, the neural net output modifies both foot placement control and stance leg control. While in the local reinforcement learning method, one neural net is trained to modify the foot placement control output, and another is trained to modify the stance leg control output. The switching between left-swing-right-stance and right-swing-left-stance is triggered by signals from the contact sensors. All the measured signals are sampled from the simulated environment. The foot placement controller converged relatively quickly in Method 1, the local reinforcement learning method, as shown in Figure 6. It needed about 15,000 walking steps and 150 iterations for a good policy to emerge, while other tasks have been reported to need 10^5^ to 10^7^ steps. Method 2, the global reinforcement learning method, needed longer trajectories in each iteration for training, and the overall convergent speed was slower than the local reinforcement learning method. Both networks were trained with a PC with a core i9 CPU. The simulation ran in real-time, and only one biped model was simulated.

## 6 Results

**FIGURE 7:**
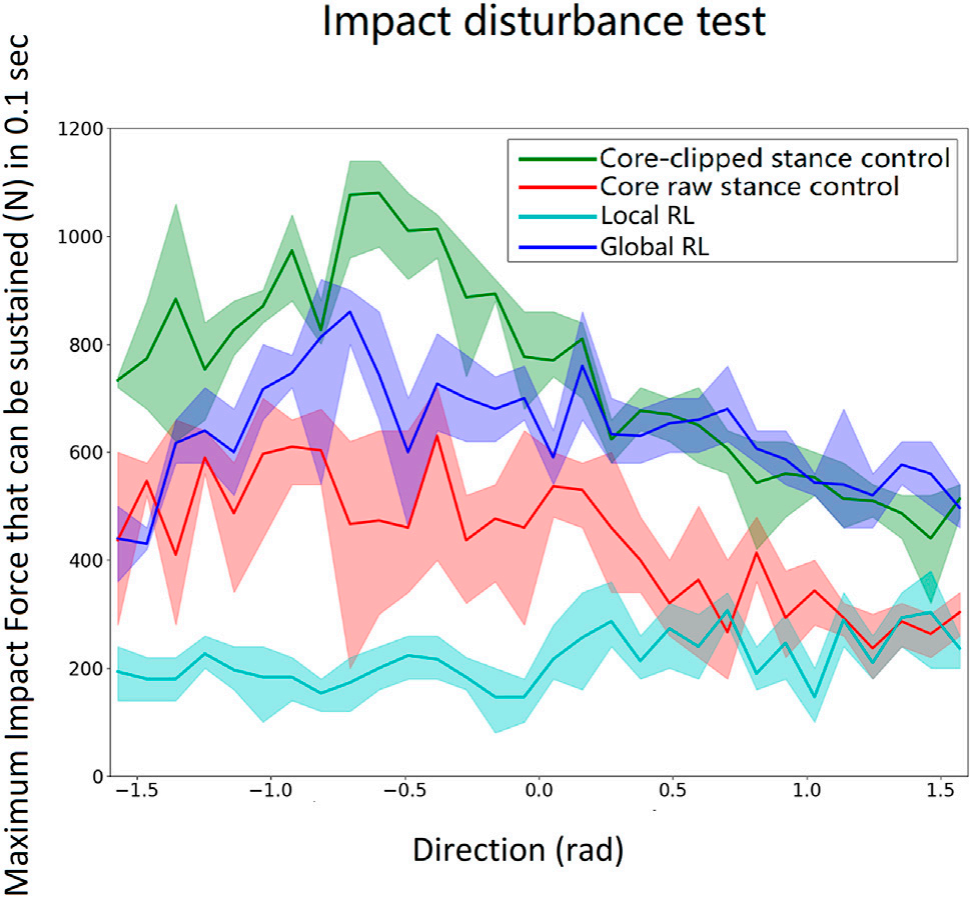
Impact load scan from − *π*/2 rad (force on the front of the torso directed rearward) to *π*/2 rad (force on the rear of the torso pushing forward) for every 0.1 rad. Each angle is tested six times. The lines indicate the mean, and the shaded regions depict the variability. The core-clipped controller has the best overall performance because it is built for the exact inertia and proportions of the model. The global local reinforcement learning method is second best and better than the raw core controller and the local local reinforcement learning method. The performance of the neural nets could be further improved if they were trained with more data. But, that would go against the concept of this work for designing a controller that can be implemented in a real-world exoskeleton with less training effort.

The robustness of the control system was tested by adding a gradually increasing short-duration (impact) force on the center of the torso during walking until the biped fell. There was a time delay between two consecutive forces to allow the biped model to return to its normal walking before the next impact. The duration of the impact force was 0.1 s. The test was performed with force applied to the torso in different directions and repeated six times for each direction. The direction of the impact force was varied from − *π*/2 rad (rearward) to *π*/2 rad (forward) for every 0.1 rad. The results for a model based on the proportions of a 1.8-m tall male human are shown in Figure 7. The result is comparable with human test by Rosenblum et al. (2020).

As can be seen in Figure 7, all four controllers (core-clipped, raw-core, global reinforcement learning method, local reinforcement learning method) generated stable gaits. However, the stable gaits generated by the neural network controllers were less robust to impact disturbances compared to the core-clipped controller.

**FIGURE 8:**
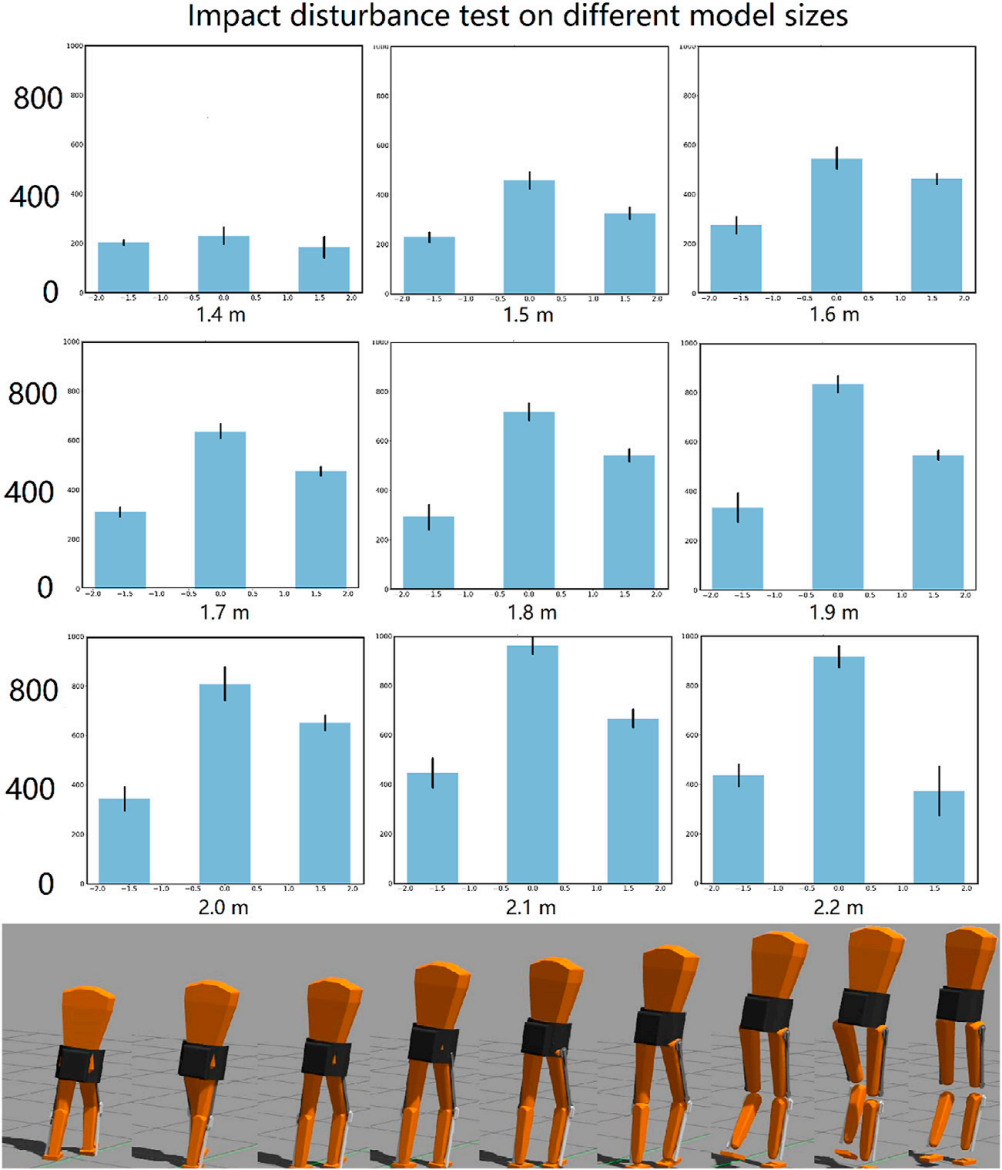
Impact disturbances test on different model sizes controlled by the same controller. In each plot (above), from left to right are the forces added in the rearward, lateral and forward direction. The different size models are shown below. Gaps between thigh and shank are because the visual mesh does not scale with the model. The moment of inertia, the mass, the link lengths, and collision models were all scaled properly.

The global neural network controller was found to be most robust to biomechanical changes in the model biped. The trained network successfully stabilized walking of biped models ranging from 1.4 to 2.2 m tall, with mass and lengths proportioned according to human data statistics (Yu-Cheng et al., 2004). Nothing in the control system was re-tuned for these very different models. The controller was trained for a 1.8-m model. The impact test was repeated for different sized models, as shown in Figure 8. Force was applied in three different directions: forward, rearward and lateral. The impact force was increased until the biped fell.

The impact robustness increased as the total mass of the biped model increased, as shown in Figure 8. This shows a low correlation between control robustness and model size within this range. On the other hand, the core controller, although more robust when the biped is in the default size, fails if the model is changed by just 0.1 m in height.

**FIGURE 9:**
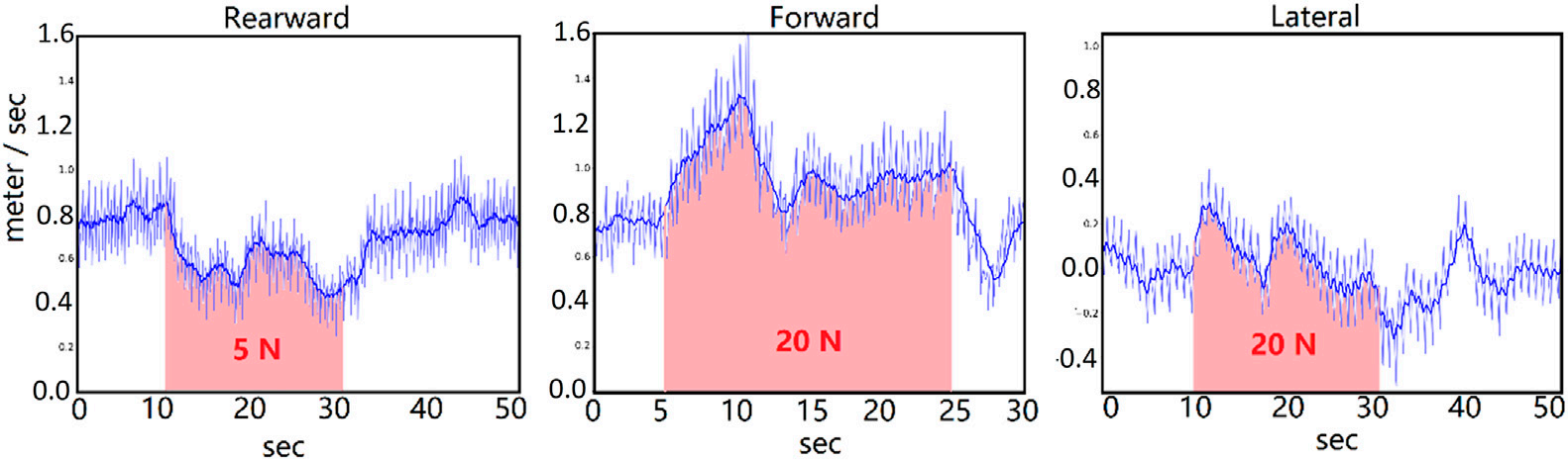
Persistent disturbance test. Speed of the biped vs. time. From left to right, the force is added rearward (pushed from the front toward the rear), forward (pushed from the rear toward the front), and laterally. In the left and middle, the speed is shown in the forward direction. In the right plot, the speed is the lateral speed in the direction of the applied force. This speed is ideally zero. As one would expect, a rearward force slows the biped, while a forward force causes it to walk faster. The controller adapts to stabilize the speed.

Next, the performance of the system was tested when a constant force was added for a long period of time. This tested the system’s robustness against persistent disturbances such as wind. The force was added on the center of the torso link for 20 s. The global neural net controller can stabilize much larger persistent forces in the forward and lateral directions compared to the rearward direction. The velocity of the biped was decreased by the rearward force. The forward force caused a short time of increasing speed, but the controller adapted to slow the pace. After the force was cleared, the biped took about 5 s to recover to normal walking. The lateral force caused little change of the lateral speed because the neural net was trained to walk forward. So, the lateral error caused by the disturbance force was compensated by the controller. This result is shown in Figure 9.

**FIGURE 10:**
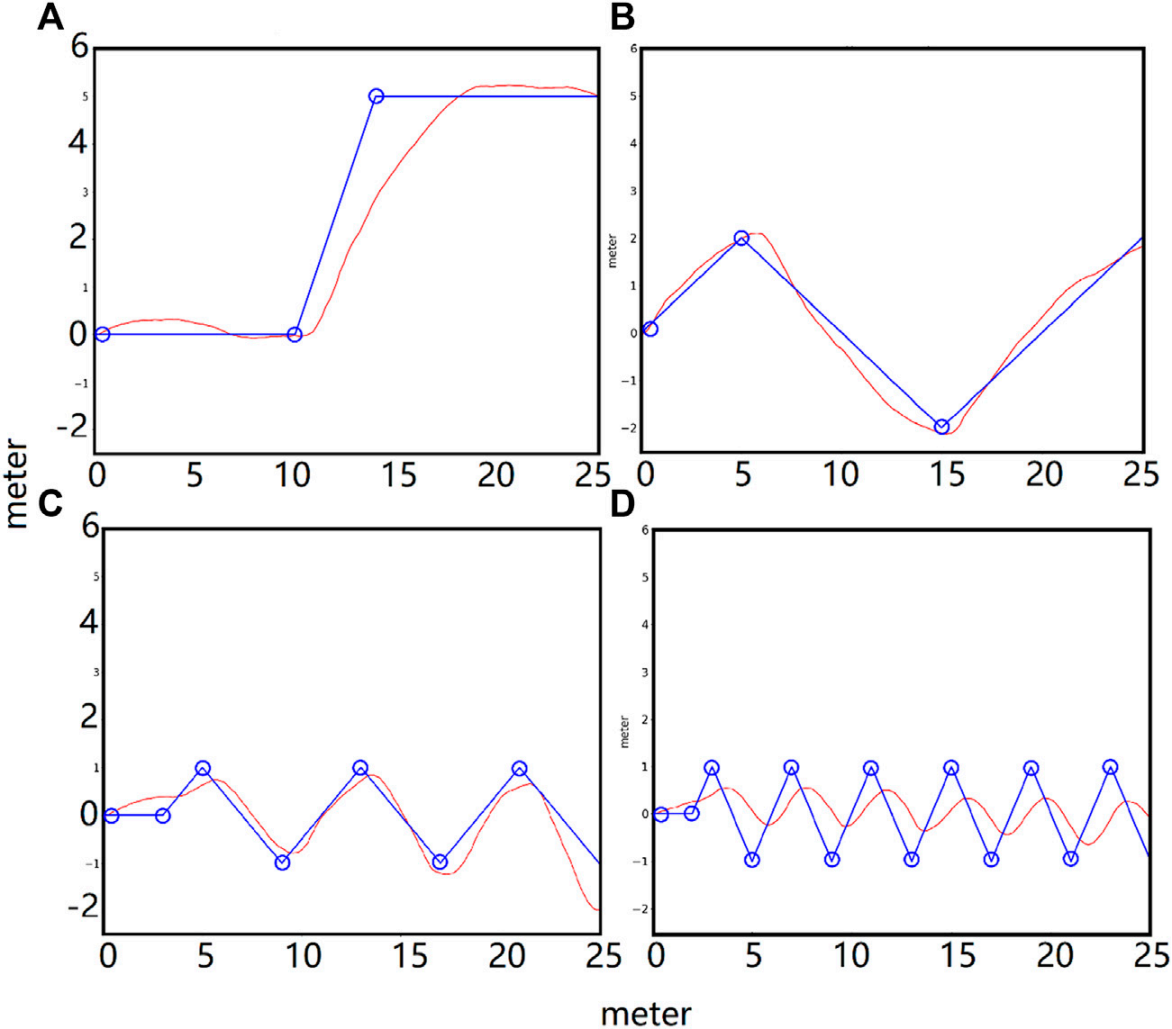
Steering tests. P lots of the path of the waypoints (blue circle) and the actual path of the torso center (red) in the x-y plane. **(A)**: step waypoint path. **(B)**: low-frequency waypoint path. **(C)**: medium-frequency waypoint path. **(D)**: high-frequency waypoint path. The static error of the waypoint following was less than 0.3 m and the lag in low-frequency turn following was small. The lag increased with higher frequency turns.

The simulated biped can turn volitionally and follow waypoints. This is achieved by adding a target coordinate error term in the environmental reward function during training. Figure 10 shows plots of the path of waypoints (blue circles) and the actual path of the torso center (red) in the x-y plane. The goal of this exercise was for the biped to walk in the x-y plane and follow the blue path. The static path error was less than 0.3 m, and the lag in low-frequency turn following was small, but this lag increased with higher frequency turns.

The neural network for the PPO policy and critic networks each have three densely connected layers with 400 units per layer. The activation function is Leaky-ReLu. Other parameters are listed in Table 2. (We use the clipped surrogate objective version in this work.) The reward function used in the waypoint-following task is as follows:

$$r=\min\left(z_{\text{torso }},1.2\right)-\frac{{\left(x_{\text{torso }}-x_{\text{target }}\right)}^{2}+{\left(y_{\text{torso }}-y_{\text{target }}\right)}^{2}}{\overset{2}{\underset{\text{target }}{y}}+x_{\text{target }}^{2}}-0.05\left(\sqrt{\overset{2}{\underset{x}{v}}+v_{y}^{2}}-v_{\text{target }}\right)$$ (28)

**TABLE 2:** neural network parameters.

Parameter	Value
Actor learning rate	1e-5
Critic learning rate	1e-4
Critic loss type	L1
Batch size	512
Num of batches	100
Max episode steps	32,000
Gamma	0.995
GAE-lambda	0.95
PPO-epsilon	0.2

A random target coordinate is generated at the beginning of each walking trial. The trial is reset when the height of the torso is lower than 0.6 m, or the y position of the torso is larger than the maximum y (+y is the forward direction). When the torso y position is larger than the current target y position, a new random target coordinate is generated.

## 7 Discussion

The results show that the core-clipped controller, developed using classical control methods, is more robust to external perturbations than the reinforcement learning policy. However, the core-clipped controller is brittle to even small changes in the plant. For example, if the user picks up a bag of groceries or if a different user dons the exoskeleton, the core-clipped controller will have to be re-tuned. This is unacceptable for an exoskeleton.

Instead of using the core controller alone, we used the core controller’s outputs and the states of the biped as the inputs to a neural network and then trained the network using reinforcement learning. The trained neural network was then shown to control stable walking of the biped. We found this method has three advantages. First, because the core controller provided a good initial solution, the network converged relatively rapidly compared to results reported in the literature. Second, the NN controller produced stable walking of the three-dimensional biped that was robust to external perturbations and drastic changes in the biomechanical model. Third, additional goals (target coordinate tracking, target speed tracking, etc.) for the controller were able to be realized because of the flexible choice of different reward functions for the RL training process.

The global reinforcement learning policy shows superior performance to the local reinforcement learning policy. In the global reinforcement learning policy, stance and swing are learned in parallel, whereas in the local reinforcement learning policy, stance and swing are learned separately. Thus, the local reinforcement learning policy is the sum of two smaller problems and converges much more rapidly.

This method is attractive for use in exoskeleton control because it does not require a detailed dynamic model of the particular user. In future work, the joint control signal can be transformed into muscle/motor activation for muscle-first driven hybrid exoskeletons (Nandor et al., 2021).

We can use this method to evaluate the efficacy of additional joint degrees of freedom to an existing exoskeleton. Adding degrees of freedom to an exoskeleton is done with care because of the added weight, complexity, and cost. Most exoskeleton’s confine the user to leg movements in the sagittal plane, which has drawbacks in terms of normal joint movements, walking speed, and stability. Additional joints can be easily added to or subtracted from a dynamic model and this method can be used to learn a controller. The performance of the system with the additional joints in terms of speed, stability, and robustness to perturbations and changes to the mechanical model (different users and carrying objects) can be evaluated. Thus, the method described in this paper can be an important tool for design as well as system control.

Shafiee-Ashtiani et al. (2017) presented improved results relative to previous walking pattern generators. In their work the controller resisted an impact force of 260 N frontal and 220 N sagittal (340 N in total) for 0.1 s in the simulation. The model weight is 98 kg. Our method can withstand 300–1000 N impact force for 0.1 s for a 1.8 m tall, 75 kg model. The training needed to achieve this performance is approximately 100 iterations or 3,200,000 steps with our setup. The results in Castillo et al. (2020) showed 800 iterations are needed. Peng et al. (2017) performed more tasks, but it required 10e5 to 10e6 iterations to train.

## 8 Conclusion

In this work, we first used classical control methods to design a core control system consisting of a stance-leg torso stabilization controller and a swing-leg foot placement controller. The stance-leg controller was based on a double pendulum model. The acceleration of the torso angle was designed to mimic a stable second-order system. Then the required torque was calculated by using double pendulum dynamics. The output was then modified to further stabilize the system. After that, the swing leg foot placement prediction controller was designed using the modified orbital energy method. The resulting “core” controller generated stable walking and was robust to impact disturbances. But it cannot stabilize a biped with different biomechanics (or if the user picks up heavy objects) because the controller was designed on precise model data. Thus, we used reinforcement learning to train a neural network for biped walking using the outputs of the core controller and the states of the biped as its inputs. Two different ways of training the policy were tested. The local reinforcement learning method used separate neural nets for swing leg control and stance leg control so that each of these neural nets only controls a portion of the action and can be trained with less computational cost. The global reinforcement learning method used just one neural net for the entire action control of the system. Results show that the local reinforcement learning method indeed trained faster than the global one, but it is less robust than the global reinforcement learning method. As the global method allows us to train the biped motion all at once, we can add additional objectives for the learning algorithms such as waypoints following. The result shows that the global neural network learns a generalized control policy that can control different sized biped models without changing any parameters. Thus, it can be used for different people with different body mass and proportions and/or for a person who dons a backpack or carries a heavy bag without the need for re-tuning. This controller also can adapt its speed to overcome persistent forces and steers to track waypoints.

## Data Availability

The original contributions presented in the study are included in the article/Supplementary Material, further inquiries can be directed to the corresponding author.
